# Raman study of flash-lamp annealed aqueous Cu_2_ZnSnS_4_ nanocrystals

**DOI:** 10.3762/bjnano.10.20

**Published:** 2019-01-17

**Authors:** Yevhenii Havryliuk, Oleksandr Selyshchev, Mykhailo Valakh, Alexandra Raevskaya, Oleksandr Stroyuk, Constance Schmidt, Volodymyr Dzhagan, Dietrich R T Zahn

**Affiliations:** 1 V. E. Lashkaryov Institute of Semiconductors Physics, National Academy of Sciences of Ukraine, Kyiv, 03028, Ukraine; 2 Semiconductor Physics, Chemnitz University of Technology, 09107 Chemnitz, Germany; 3 L. V. Pysarzhevsky Institute of Physical Chemistry, National Academy of Sciences of Ukraine, Kyiv, 03028, Ukraine

**Keywords:** copper zinc tin sulfide Cu_2_ZnSnS_4_ (CZTS), CuS, Cu-Sn-S, kesterite, phonon, pulsed light crystallization, Raman spectroscopy, secondary phase, SnS

## Abstract

The effect of flash-lamp annealing (FLA) on the re-crystallization of thin films made of colloidal Cu_2_ZnSnS_4_ nanocrystals (NCs) is investigated by Raman spectroscopy. Unlike similar previous studies of NCs synthesized at high temperatures in organic solvents, NCs in this work, which have diameters as small as 2–6 nm, were synthesized under environmentally friendly conditions in aqueous solution using small molecules as stabilizers. We establish the range of FLA conditions providing an efficient re-crystallization in the thin film of NCs, while preserving their kesterite structure and improving their crystallinity remarkably. The formation of secondary phases at higher FLA power densities, as well as the dependence of the formation on the film thickness are also investigated. Importantly, no inert atmosphere for the FLA treatment of the NCs is required, which makes this technology even more suitable for mass production, in particular for printed thin films on flexible substrates.

## Introduction

Affordable and non-toxic solar energy materials having a high absorption coefficient and a bandgap in the solar illumination range are an ever-growing research field. Among them are kesterite Cu_2_ZnSnS_4_ (CZTS) and related compounds [1–3]. This class of compounds can be used in the promising technology of “printing” solar cells using inks based on CZTS nanocrystals (NCs) [4–7].

However, the structural complexity of these compounds results in numerous structural defects and secondary phases of binary and ternary compounds of the constituent elements, such as copper and tin sulfides [8–13]. Therefore, the issue of preserving and improving the quality of the ink NC material during application-relevant film formation processes is of paramount importance. The technologies of rapid annealing by thermal heating or intensive pulsed light radiation turned out to be very promising [14–16]. The effectiveness of the method of flash-lamp annealing (FLA) was demonstrated for various compounds, including quaternary metal chalcogenides [17–18]. The latter was proposed by the authors of [18] for processing CZTS in the production of solar cells. For colloidal CZTS NCs FLA was applied only in [19]. However, the NCs employed in the latter work were synthesized (using the protocol from [20]) at high temperatures around 340 °C and had a relatively large size of 35 nm. The effect of annealing was investigated only up to an energy density of 11.6 J/cm^2^ and possible influences of NC film thickness and ambient atmosphere were not considered.

In this work, we investigated the effect of FLA on CZTS NCs synthesized under mild conditions, namely at 80 °C in aqueous solution using thioglycolic (mercaptoacetic) acid as a stabilizer. The NCs have a size of 2–6 nm [21], which is much smaller than in [19]. Therefore, the effect of the FLA on these significantly different NCs can be very distinct.

We investigated the effect of various doses of irradiation energy, up to 60 J/cm^2^, on NC films deposited on a glass substrate by drop-casting. The influence of film thickness and crystallinity of the initial NCs (before FLA) was also studied. Raman scattering was chosen as a main characterization method in this work, because it has already proved to be very efficient diagnostic tool of the structure and composition of CZTS and related compounds [10–11,13,22–30]. Moreover, being a fast, contactless, and non-destructive technique, Raman spectroscopy/microscopy can be applied for in situ monitoring in CZTS film technology, as it serves for decades in Si microelectronics [31] and CIGS solar cell technology [32–33].

## Results and Discussion

### Characterization of original CZTS NC films

The first observation made on the difference between the samples ink0 and ink1 was an unequal morphology of the films formed. Those formed from ink0 were rather homogeneous, while in case of ink1 the films showed a more pronounced “coffee ring” morphology, with thinner spots in the center of the drop and thicker ones on its edges (see Supporting Information File 1, Figure S1). We assume that the better film-forming properties of ink0 are due to the larger content of the ligand (according to XPS, 27% O and 40% C in ink0 vs 22% O and 37% C in ink1) or/and probably some structural and compositional changes of the organic matrix initiated through the heat treatment of ink1.

The elemental composition of the CZTS NC phase was proved by a survey XPS analysis, that confirmed the presence of all the expected elements (Cu, Zn, Sn, S), while high-resolution core-level spectra were acquired to prove the oxidation state expected for the CZTS compound (Supporting Information File 1, Figure S2).

The Raman spectra presented in the manuscript were acquired with an excitation laser power of 0.1 mW (10^6^ W/cm^2^). We also investigated the spectra at laser powers down to 0.001 mW (10^4^ W/cm^2^) to ensure that the material was not notably modified during the Raman measurements (see Supporting Information File 1, Figure S4 and Figure 6 in [34]). An additional proof that the laser power does not affect the samples are our recently reported low-temperature measurements (77 K) [34], which revealed no qualitative changes in the spectra except for an expected temperature-induced shift and narrowing of the phonon peaks.

**Figure 1:**
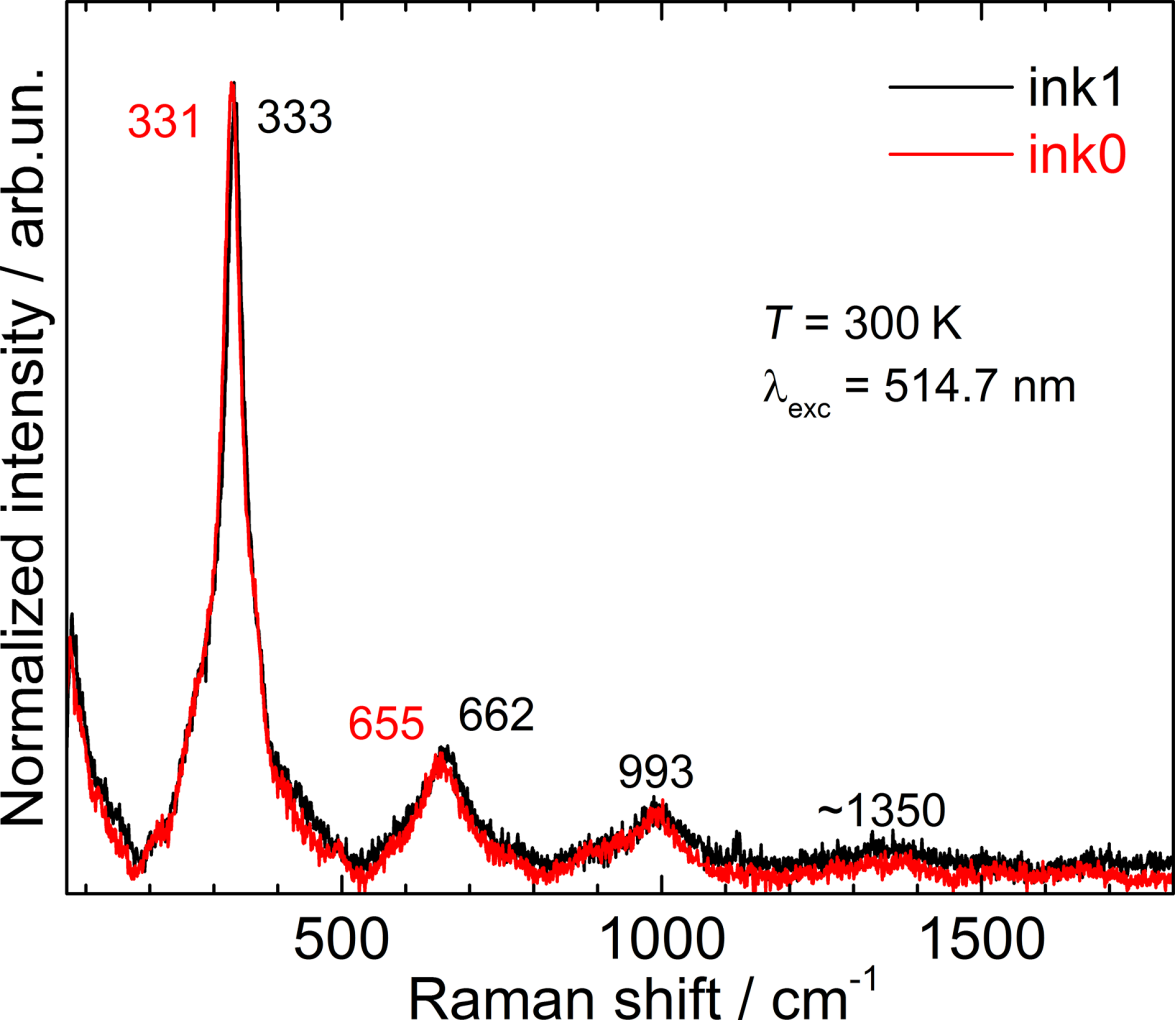
Raman spectra of ink0 and ink1 NC films before FLA treatment.

The Raman spectra of the ink1-derived films appeared to be spectrally homogeneous and revealed a strong phonon peak at 333 cm^−1^ when measured in different spots of the films (Figure 1). In contrast, despite the optically homogeneous surface, the spectra of ink0-derived films showed a variation of the peak position between 328 and 331 cm^−1^ over the sample area. From the same FWHM of Raman bands of both inks we can conclude that the degree of crystallinity is not significantly affected by the post-synthesis heating of the NC solution. The spatial inhomogeneity of the films formed of pristine NCs (ink0) can be considered as an indication to a more probable nucleation of secondary phases as described in more detail in the following discussion.

In a separate detailed investigation of the influence of film deposition and drying conditions on the formation of secondary phases we found the optimum substrate and environment for significantly reducing the secondary-phase content [34]. In the present study, neither of the two NC sample types shows Raman peaks of phases other than CZTS. Moreover, the series of overtones, up to the fifth order (ca. 1700 cm^−1^), in the spectra of both inks (Figure 1) is a further proof of their high crystallinity. It should be mentioned that even though the frequencies of the main Raman peak in the range of (331–334) cm^−1^ were often reported for CZTS NCs identified as kesterite (KS) [35–38], the frequency of this phonon mode in a perfect bulk KS CZTS was established to be 337–339 cm^−1^ [39–41]. The reasons for the lower frequency values can be cation disorder [10,39], the formation of other polytypes, particularly so-called disordered KS [10,39], stannite [39,41], and wurtzite (WZ) [42–44]. Based on the XRD data of our samples [21], we can exclude the WZ phase. Furthermore, the Raman spectra of freshly synthesized solutions indeed showed peaks at 337–339 cm^−1^, i.e., the kesterite bulk frequencies, but the quality of the Raman spectra was very poor because of a strong photoluminescence (PL) background (Supporting Information File 1, Figure S3).

In the spectra of the dried films, the PL background, most likely related to minor organic byproducts of the CZTS NCs synthesis reaction, was absent, allowing for the recording of the high-quality Raman spectra. However, the position of the main peak was observed at about 5 cm^−1^ lower frequency (Figure 1). There are two possible reasons of the observed frequency shift in the films (as well as for the solutions stored for longer times): (i) rearrangement of cations, possibly stimulated by a reaction with oxygen and accompanied by a change of the charge state of Cu from +1 to +2; or (ii) re-crystallization of the NCs into larger NCs while preserving the crystal structure. A good correlation of the NC size obtained from UV–vis spectra (on fresh solutions) and from XRD (on films) reported in [21] seems to rule out reason (ii) as a major contributor. Therefore, most likely there are some structural changes in the cationic sublattice, similar to what we reported for bulk CZTS [10–11]. The discussed “disorder” does not mean, however, the absence of crystallinity, as can be concluded from the sharp phonon spectra (Figure 1). It is rather a certain rearrangement of the cations (and probably anions) in the sublattice that does not deteriorate the overall crystallinity of the NC.

### Flash-lamp annealing procedures

FLA was carried out in air and in nitrogen atmosphere, but no significant effect of the atmosphere on the quality of the films was observed in the Raman spectra. The formation of secondary phases, both in air and in N_2_, started at energy densities of 10–15 J/cm^2^, and above 15 J/cm^2^ the difference between the annealing atmospheres was observed only in a slightly different surface morphology for ink1-derived films.

**Figure 2:**
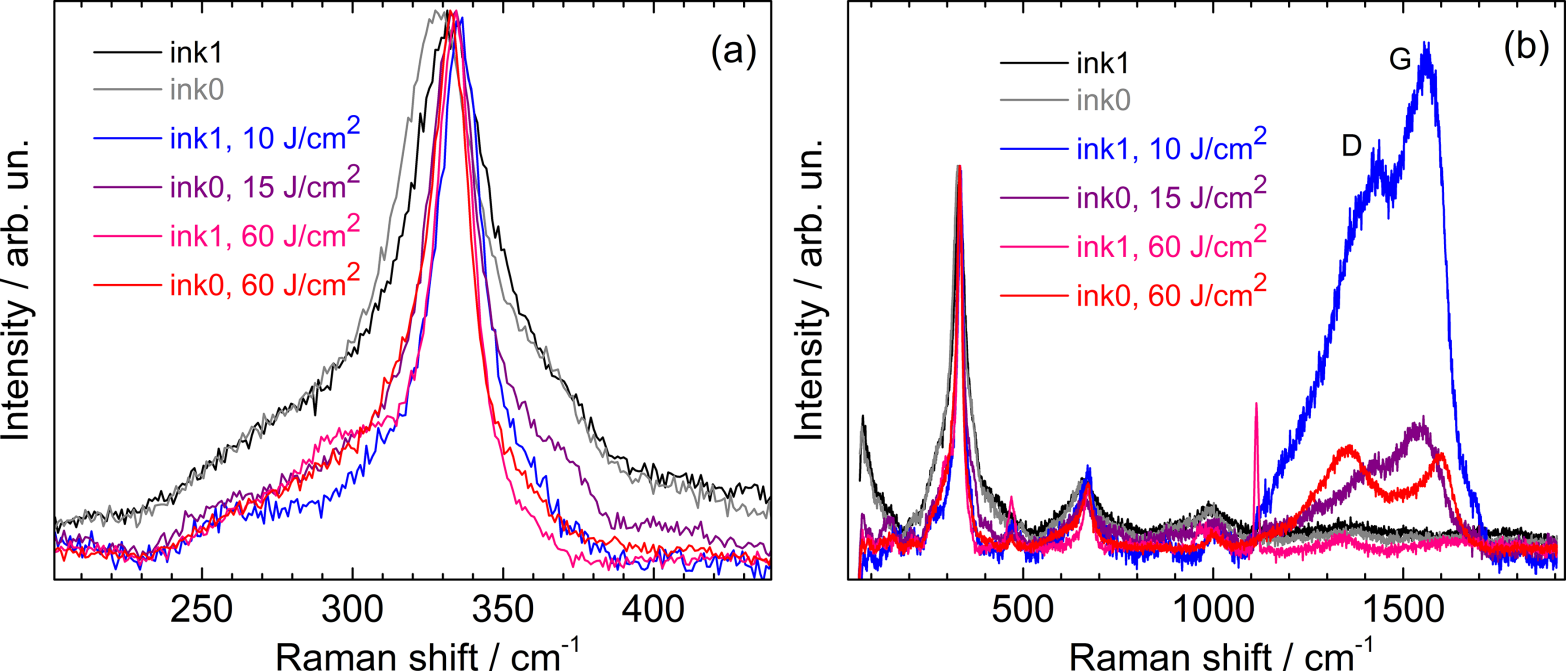
Raman spectra of ink0 and ink1 NC films before and after FLA at different power densities: (a) spectral range of the main CZTS phonon mode, (b) in the broad range including carbon D- and G-bands.

At 5–10 J/cm^2^, crystallization of the initial NC film occurred, as can be inferred from a shift of the main Raman peak towards higher frequencies, its narrowing and the appearance of additional CZTS peaks in the range of 250–300 cm^−1^ (Figure 2a). The sample surface remained optically and spectrally homogeneous in this case.

At 25–35 J/cm^2^, there was a significant difference between ink0 and ink1. For ink1, the increase of the power density led to the decay of the CZTS phase in the central, thinner part of the film into secondary phases. But at the edge of the film, i.e., in the part of the thickest “coffee rings”, the spectra obtained correspond to crystalline bulk-like material. For ink0, under the same FLA conditions, there was a more homogeneous phase distribution over the surface with approximately the same area of the secondary phases and the CZTS material. The results obtained allow us to conclude that the quality of the flash annealed aqueous CZTS NCs of small size is expectably determined not only by the energy density, but also by the thickness of the film. Thus, in the case of annealing of thin films with high energies, the Raman spectra show the presence of all possible secondary phases and the absence of the source material, indicating a complete decay of CZTS. When thicker films are annealed, even with maximum energy, the Raman spectra correspond to the spectra of kesterite CZTS.

**Figure 3:**
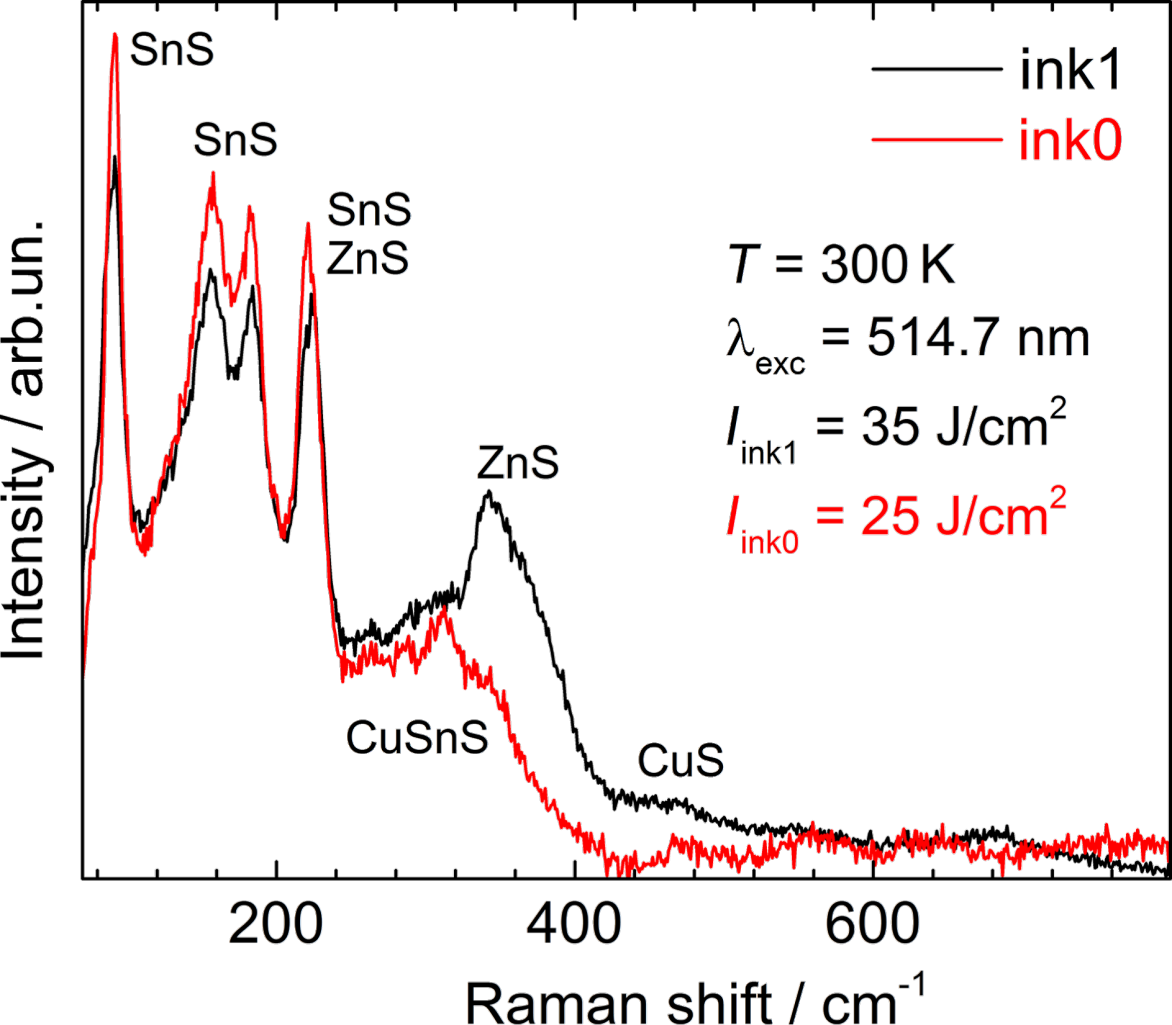
Raman spectra of ink0 (*I* = 25 J/cm^2^) and ink1 (*I* = 35 J/cm^2^) NC films with the most pronounced decomposition into secondary phases.

After the highest content of secondary phases for ink0 was observed at 25 J/cm^2^ and for ink1 at 30–35 J/cm^2^ (Figure 3), it was reduced significantly after further increase of the annealing energy densities, e.g., at 60 J/cm^2^. There is a small peak of Cu_2−_*_x_*S detected in the latter case (Figure 2b), but the quality of the CZTS spectra is close to that of films treated at 10–15 J/cm^2^ (Figure 2a). From this observation, we can assume that probably the secondary phases created by FLA (presumably on the surface) are also removed by it, leaving the re-crystallized CZTS material.

Another interesting fact is that in the ink1 sample with the highest crystallization observed at 15 J/cm^2^, the position of the main peak in the Raman spectra was 337 cm^−1^, which almost coincides with the peak position for bulk crystalline CZTS 337–339 cm^−1^ [39–41]. At the same time, for ink0, the highest Raman peak frequency measured after FLA treatment did not exceed 334 cm^−1^. Apparently, the structural changes achieved by the post-synthesis treatment in solution are of critical importance for the subsequent modifications through FLA.

In all the FLA-treated films, broad Raman features in the range of carbon-related D- and G-bands were detected (Figure 2b). These bands are related to the decomposition of ligands into amorphous carbon phases [45]. No relation between the intensity if these bands and the quality of the CZTS spectrum was observed. The carbon bands are observed both in spectra with only CZTS peaks as well as in those in which only secondary phases are detected.

## Conclusion

We investigated the effect of flash-lamp annealing on small CZTS NCs synthesized under mild conditions in aqueous solution, using small molecules as stabilizers. We found that successful re-crystallization of a NC film can be achieved at FLA power densities of 5–10 J/cm^2^. In the range between 10 and 60 J/cm^2^ an extensive decomposition of CZTS into binary and ternary phases dominates over the improvement of the crystal quality of the CZTS phase. Surprisingly, at 60 J/cm^2^ a high-quality CZTS signal could be observed again. This is probably related to the removal/evaporation of the secondary phases during FLA. Importantly, no notable influence of the inert atmosphere was detected. Therefore, the low-power FLA crystallization, which turned out to be successful, can be performed under ambient conditions, making this procedure even more suitable for mass production.

## Experimental

The synthesis of the CZTS NCs used in this work and the basic characterization by UV–vis, XRD, Raman spectroscopy and TEM were reported by us previously [21]. Shortly, the CZTS NCs were prepared in aqueous solutions by the reaction of a mixture of mercaptoacetate (MA) complexes of Cu(II), Sn(II), and Zn(II) with sodium sulfide and a subsequent optional heat treatment at about 95 °C. An XPS study reported in [21] showed all the elements of CZTS to be present in the expected valence states (Cu+, Zn^2+^, Sn^4+^, S^2−^), Cu(II) was reduced to Cu(I) and Sn(II) was oxidized to Sn(IV) in situ during the NC formation. The concentration of the as-prepared CZTS colloids was around 30 g/L and can be further increased by solvent evaporation or a precipitation/redispersion procedure [21].

Here, we focus on the effect of flash-lamp annealing on the structural evolution of CZTS films, as probed by Raman spectroscopy. The FLA setup, based on a xenon lamp emitting in the range of 300 to 800 nm, was customized by Dresden Thin Film (DTF) Technology GmbH [33] in a glove box system with nitrogen atmosphere. Two types of samples were investigated: an as-synthesized sample “ink0” (the sample purified without heat treatment at 95 °C) [21], and “ink1”, i.e., the same ink heated at 96–98 °C in the dark immediately after synthesis (vials were immersed for 10 min into a boiling water bath). For FLA treatment, CZTS samples were deposited on a glass substrate by drop-casting. In order to investigate the effect of flash annealing at various energy densities, a series of samples was prepared from 0 up to 60 J/cm^2^ in steps of 5 J/cm^2^ with a pulse duration of 17.8 ms.

Raman spectra were excited with the λ_exc_ = 514.7 nm line of a DPSS laser (Cobolt) and recorded at a spectral resolution of about 3 cm^−1^ using a LabRam HR800 micro-Raman system equipped with a liquid-nitrogen-cooled CCD detector. The incident laser power under the microscope objective (50×) was *P*_1_ = 0.1 mW or *P*_2_ = 0.001 mW, corresponding to about 3∙10^6^ and 3∙10^4^ W/cm^2^, respectively.

## Supporting Information

File 1 Additional experimental data.

## References

[1] Green M A, Hishikawa Y, Warta W, Dunlop E D, Levi D H, Hohl-Ebinger J, Ho-Baillie A W H (2017). Prog Photovoltaics.

[2] Wallace S K, Mitzi D B, Walsh A (2017). ACS Energy Lett.

[3] Sandroni M, Wegner K D, Aldakov D, Reiss P (2017). ACS Energy Lett.

[4] Kovalenko M V, Manna L, Cabot A, Hens Z, Talapin D V, Kagan C R, Klimov V I, Rogach A L, Reiss P, Milliron D J (2015). ACS Nano.

[5] Pietryga J M, Park Y-S, Lim J, Fidler A F, Bae W K, Brovelli S, Klimov V I (2016). Chem Rev.

[6] Freitas J N, Gonçalves A S, Nogueira A F (2014). Nanoscale.

[7] Zhou M, Gong Y, Xu J, Fang G, Xu Q, Dong J (2013). J Alloys Compd.

[8] Kumar M, Dubey A, Adhikari N, Venkatesan S, Qiao Q (2015). Energy Environ Sci.

[9] Baranowski L L, Zawadzki P, Lany S, Toberer E S, Zakutayev A (2016). Semicond Sci Technol.

[10] Valakh M Y, Dzhagan V M, Babichuk I S, Fontane X, Perez-Rodriquez A, Schorr S (2013). JETP Lett.

[11] Valakh M Y, Kolomys O F, Ponomaryov S S, Yukhymchuk V O, Babichuk I S, Izquierdo-Roca V, Saucedo E, Perez-Rodriguez A, Morante J R, Schorr S (2013). Phys Status Solidi RRL.

[12] Tan J M R, Lee Y H, Pedireddy S, Baikie T, Ling X Y, Wong L H (2014). J Am Chem Soc.

[13] Dimitrievska M, Boero F, Litvinchuk A P, Delsante S, Borzone G, Perez-Rodriguez A, Izquierdo-Roca V (2017). Inorg Chem.

[14] Prucnal S, Gao K, Zhou S, Wu J, Cai H, Gordan O D, Zahn D R T, Larkin G, Helm M, Skorupa W (2014). Appl Phys Lett.

[15] Büchter B, Seidel F, Fritzsche R, Toader I, Buschbeck R, Jakob A, Schulze S, Freitag H, Lang H, Hietschold M (2014). J Mater Sci.

[16] Büchter B, Seidel F, Fritzsche R, Lehmann D, Bülz D, Buschbeck R, Jakob A, Schulze S, Freitag H, Lang H (2015). J Mater Sci.

[17] Druffel T, Dharmadasa R, Lavery B W, Ankireddy K (2018). Sol Energy Mater Sol Cells.

[18] Munn C, Haran S, Seok I (2013). Proc SPIE.

[19] Williams B A, Smeaton M A, Holgate C S, Trejo N D, Francis L F, Aydil E S (2016). J Vac Sci Technol, A.

[20] Chernomordik B D, Béland A E, Trejo N D, Gunawan A A, Deng D D, Mkhoyan K A, Aydil E S (2014). J Mater Chem A.

[21] Stroyuk O, Raevskaya A, Selyshchev O, Dzhagan V, Gaponik N, Zahn D R T, Eychmüller A (2018). Sci Rep.

[22] Khare A, Himmetoglu B, Johnson M, Norris D J, Cococcioni M, Aydil E S (2012). J Appl Phys.

[23] Grosvenor A P, Biesinger M C, Smart R S C, McIntyre N S (2006). Surf Sci.

[24] Fernandes P A, Salomé P M P, da Cunha A F (2011). J Alloys Compd.

[25] Dimitrievska M, Fairbrother A, Pérez-Rodríguez A, Saucedo E, Izquierdo-Roca V (2014). Acta Mater.

[26] Valakh M Y, Litvinchuk A P, Dzhagan V M, Yukhymchuk V O, Yaremko A M, Romanyuk Y A, Guc M, Bodnar I V, Pérez-Rodríguez A, Zahn D R T (2016). J Phys: Condens Matter.

[27] Litvinchuk A P, Dzhagan V M, Yukhymchuk V O, Valakh M Y, Parasyuk O V, Piskach L V, Wang X, Jacobson A J, Zahn D R T (2016). Phys Status Solidi B.

[28] Litvinchuk A P, Dzhagan V M, Yukhymchuk V O, Valakh M Y, Babichuk I S, Parasyuk O V, Piskach L V, Gordan O D, Zahn D R T (2014). Phys Rev B.

[29] Valakh M Y, Litvinchuk A P, Dzhagan V M, Yukhymchuk V O, Havryliuk Y O, Guc M, Bodnar I V, Izquierdo-Roca V, Pérez-Rodríguez A, Zahn D R T (2016). RSC Adv.

[30] Dimitrievska M, Fairbrother A, Fontané X, Jawhari T, Izquierdo-Roca V, Saucedo E, Pérez-Rodríguez A (2014). Appl Phys Lett.

[31] Wolf I D (1996). Semicond Sci Technol.

[32] Scheer R, Pérez-Rodríguez A, Metzger W K (2010). Prog Photovoltaics.

[33] (2018). FLA und PLA 2 - Helmholtz-Zentrum Dresden-Rossendorf, HZDR.

[34] Havryliuk Y, Valakh M Y, Dzhagan V, Greshchuk O, Yukhymchuk V, Raevskaya A, Stroyuk O, Selyshchev O, Gaponik N, Zahn D R T (2018). RSC Adv.

[35] Zou C, Zhang L, Lin D, Yang Y, Li Q, Xu X, Chen X, Huang S (2011). CrystEngComm.

[36] Jiang H, Dai P, Feng Z, Fan W, Zhan J (2012). J Mater Chem.

[37] Flynn B, Wang W, Chang C-h, Herman G S (2012). Phys Status Solidi A.

[38] Park J, Song M, Jung W M, Lee W Y, Kim H, Kim Y, Hwang C, Shim I-W (2013). Dalton Trans.

[39] Huang S, Luo W, Zou Z (2013). J Phys D: Appl Phys.

[40] Babichuk I S, Yukhymchuk V O, Dzhagan V M, Valakh M Y, Leon M, Yanchuk I B, Gule E G, Greshchuk O M (2013). Funct Mater.

[41] Ge J, Yu W, Cao H, Jiang J, Ma J, Yang L, Yang P, Hu Z, Chu J (2012). Phys Status Solidi A.

[42] Mainz R, Singh A, Levcenko S, Klaus M, Genzel C, Ryan K M, Unold T (2014). Nat Commun.

[43] Singh A, Singh S, Levcenko S, Unold T, Laffir F, Ryan K M (2013). Angew Chem, Int Ed.

[44] Singh A, Geaney H, Laffir F, Ryan K M (2012). J Am Chem Soc.

[45] Ferrari A C, Robertson J (2000). Phys Rev B.

