# Supplementary material for: Raman study of flash-lamp annealed aqueous Cu2ZnSnS4 nanocrystals
Source: Beilstein J Nanotechnol. 2019 Jan 17;10:222–7. doi: 10.3762/bjnano.10.20 (PMC6350886; doi:10.3762/bjnano.10.20)
Supplement: File 1 — Additional experimental data. [file Beilstein_J_Nanotechnol-10-222-s001.pdf]

BEILSTEIN JOURNAL OF NANOTECHNOLOGY

Supporting Information

for

Raman study of flash-lamp annealed aqueous Cu_2ZnSnS_4 nanocrystals

Yevhenii Havryliuk, Oleksandr Selyshchev, Mykhailo Valakh, Alexandra Raevskaya, Oleksandr Stroyuk, Constance Schmidt, Volodymyr Dzhagan and Dietrich R. T. Zahn

Beilstein J. Nanotechnol. **2019,** *10,* 222–227. doi:10.3762/bjnano.10.20

Additional experimental data

License and Terms: This is a supporting information file under the terms of the Creative Commons Attribution License (http://creativecommons.org/licenses/by/4.0). Please note that the reuse, redistribution and reproduction in particular requires that the authors and source are credited.
The license is subject to the *Beilstein Journal of Nanotechnology* terms and conditions: (https://www.beilstein-journals.org/bjnano)

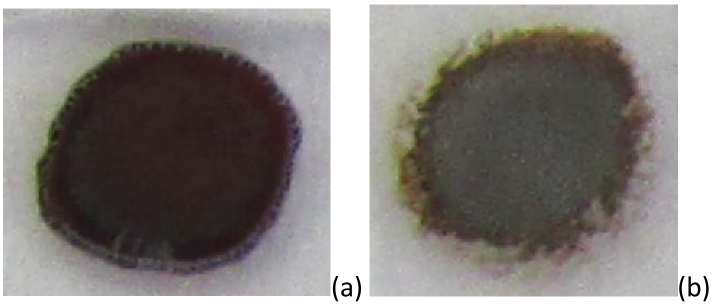

Figure S1: Optical images of the films obtained by drop-casting from ink0 (a) and ink1 (b).

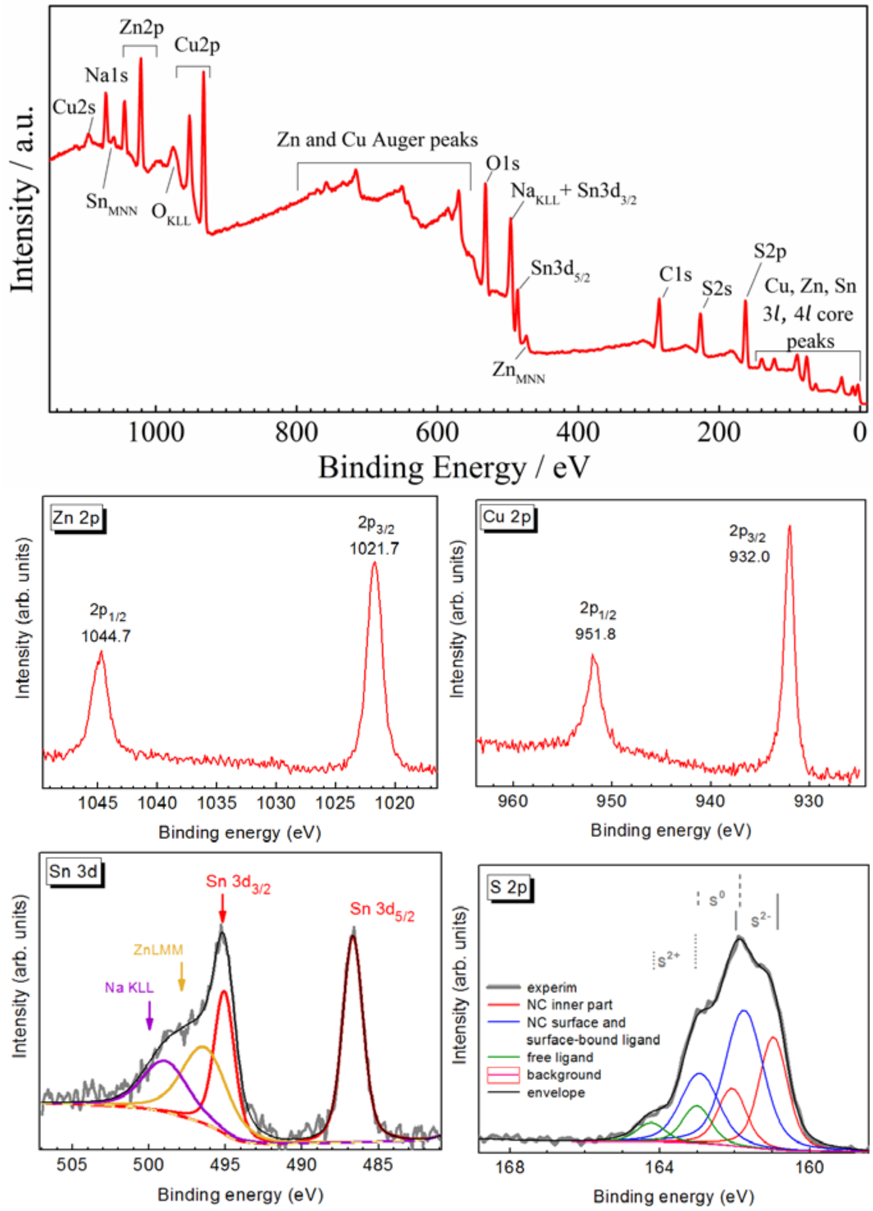

Figure S2: A representative survey XPS spectrum of CZTS NCs ink1 (top) along with high-resolution core-level spectra of Cu 2p, Zn 2p, Sn 3d, and S 2p. Deconvolution of the spectra of Sn 3d and S2p are also shown. The Zn 2p and Cu 2p are consisting of a single component corresponding to Zn^{2+} and Cu^{+}, respectively.

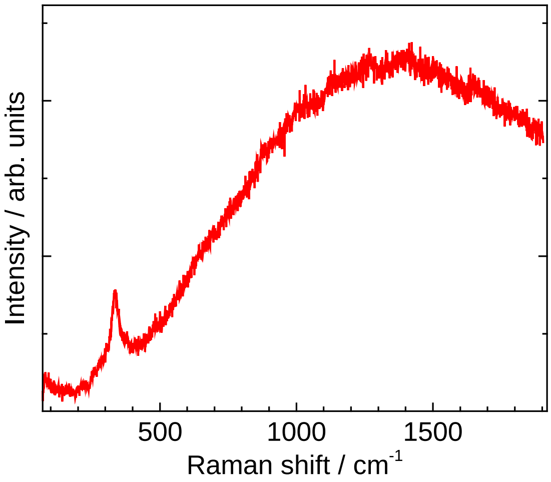

Figure S3: Representative raw Raman spectrum of the freshly prepared NCs in solution.

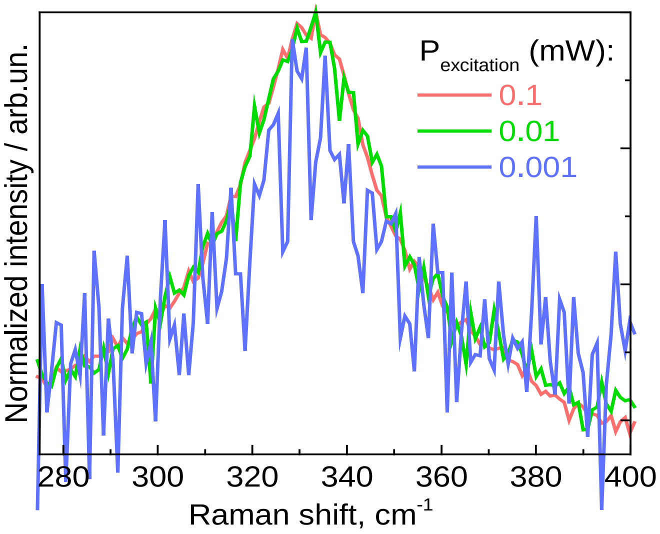

Figure S4: Representative raw Raman spectrum of the CZTS NC film measured at different powers of the 514.5 nm laser excitation.
